# Knowledge of ghostwriting and financial conflicts-of-interest reduces the perceived credibility of biomedical research

**DOI:** 10.1186/1756-0500-4-27

**Published:** 2011-01-31

**Authors:** Jeffrey R Lacasse, Jonathan Leo

**Affiliations:** 1 School of Social Work, College of Public Programs, Arizona State University, Phoenix, Arizona, USA; 2 Lincoln Memorial University - DeBusk College of Osteopathic Medicine, Harrogate, Tennessee, USA

## Abstract

**Background:**

While the impact of conflicts-of-interest (COI) is of increasing concern in academic medicine, there is little research on the reaction of practicing clinicians to the disclosure of such conflicts. We developed two research vignettes presenting a fictional antidepressant medication study, one in which the principal investigator had no COI and another in which there were multiple COI disclosed. We confirmed the face validity of the COI vignette through consultation with experts. Hospital-based clinicians were randomly assigned to read one of these two vignettes and then administered a credibility scale.

**Findings:**

Perceived credibility ratings were much lower in the COI group, with a difference of 11.00 points (31.42%) on the credibility scale total as calculated through the Mann-Whitney U test (95% CI = 6.99 - 15.00, *p *< .001). Clinicians in the COI group were also less likely to recommend the antidepressant medication discussed in the vignette (Odds Ratio = 0.163, 95% CI = .03 = 0.875).

**Conclusions:**

In this study, increased disclosure of COI resulted in lower credibility ratings.

## Background

The impact of financial conflicts-of-interest (COI) in medicine is a topic of increasing concern [1]. Several studies have found that financial COI play an important role in the presentation and interpretation of research [2,3], and that studies sponsored by industry are more likely to result in the publication of positive findings [4-6]. At present, most medical journals address the issue by requiring that authors disclose their financial COI in a competing interests statement [7].

A recent systematic review [8] finds only a few investigations of the impact of financial COI disclosure on clinicians, key consumers of medical research. In an oft-cited study, readers of *BMJ *were sent an article on the treatment of herpes zoster and randomly assigned to receive either a version in which the authors declared financial COI (as employees of a fictional pharmaceutical company) or declared no competing interests (as clinicians at an ambulatory care center). Respondents judged the version with disclosure of financial COI as less valid and believable [9]. Similar findings from other studies suggest that when financial COI are disclosed revealing that the authors have a commercial interest in the results, credibility ratings are lower [10,11]. In light of evidence that financial COI do influence the content of published research conclusions [2,4,12], these lower credibility ratings in the presence of COI may be justified.

Previous studies have tested the impact of financial COI by presenting these COI in a relatively straightforward manner (e.g., testing the inclusion of a competing interests statement accompanying a research article). However, the information disclosed in competing interests statements is limited and may omit important information. For instance, disclosure of competing interests is currently limited to listing the corporations and agencies that have paid or funded the investigator in the recent past. The amounts of payments are typically not listed. Whether a professor has received $1,000 or $400,000 for consulting with a pharmaceutical company, this financial COI will be listed in exactly the same way.

Competing interests statements also may not specify the activities for which the investigator is paid. In the recent past, there has been significant criticism of the payment and utilization of "key opinion leaders" (KOLs) [13-15]. KOLs are paid to consult with the marketing departments of pharmaceutical companies, and to deliver talks to physicians about the companies' products. Concerns have been raised about the scientific objectivity of KOLs [16].

Finally, an important issue in the management of COI is that of ghostwriting. In this instance, a medical writer (pharmaceutical company employee or subcontractor) writes a manuscript in conjunction with the marketing department of the company, and an academic researcher is listed as author [17,18]. In many cases, the actual writer of the article is not listed as an author, which amounts to an undisclosed COI, as the pharmaceutical company's involvement in the preparation of the manuscript remains unacknowledged [19,20]. Ghostwritten articles have been identified as sources of clandestine commercial influence (e.g., [21]), and some research suggests that such articles are impacting in the peer-reviewed literature [22,23]. Since it is covert by definition, ghostwriting cannot be adequately managed through the use of competing interests statements.

We sought to assess the impact of multiple types of COI (financial COI, KOL status, and ghostwriting) on the perceived credibility of biomedical research among practicing clinicians, using a vignette design providing information beyond that disclosed in a competing interests statement.

## Methods

Two vignettes were created describing a fictional study of a new antidepressant ("Serovux") for pediatric use. Both research vignettes were identical in describing the study sample, methods, and results, and in claiming that Serovux was safe and effective for children, using language derived from a well-known pediatric antidepressant study [24]. Vignettes differed only in terms of their financial COI and the method of authorship. In the non-COI vignette (323 words long), the professor presenting the research has no financial COI, is funded by the National Institute of Mental Health (NIMH), does not accept money from industry, and authors the article in collaboration with colleagues.

In the COI vignette (411 words long), the professor receives about $100,000 a year for ongoing KOL-type consulting with the maker of the antidepressant, and the manuscript is authored by a medical writer who is not listed as a co-author. The COI content was based on several known occurrences of ghostwriting, some of which have emerged through legal discovery in medico-legal cases [21,25,26]. The $100,000 COI amount was derived from science media coverage of prominent research psychiatrists receiving payments from industry [27]. These vignettes are available as supplemental files [Additional file 1 and additional file 2].

Five expert raters were asked to assess the COI vignette for face validity. We selected raters who were academic researchers and who had published peer-reviewed journal articles and/or scholarly books on ghostwriting. Three of the five raters had served as medico-legal experts in litigation involving ghostwritten journal articles. These expert raters opined that our vignettes had a high level of face validity (see Table 1), and that the vignette accurately reflected ghostwriting incidents known to have occurred in real life.

**Table 1:** Assessment of face validity by ghostwriting experts

Statement	Level of Agreement (n = 5)*	Percent of Agreement
"Dr. Harvey is a Key Opinion Leader"	4.0 ±1.73**†**	80%

"The vignette accurately describes an incident of ghostwriting similar to those known to have occurred in real life."	4.6 ± 0.548	100%

"A psychiatrist who reads the antidepressant RCT literature is likely to come across studies that were generated in a manner similar to that described in the vignette." **‡**	4.6 ± 0.548	100%

"The multiple conflicts-of-interest described in this vignette have been common among authors of RCTs in the SSRI and SNRI literature."	4.8 ± 0.447	100%

* Responses were on a 5-point Likert-type scale, with 1 = Strongly Disagree, 2 = Disagree, 3 = Neutral, 4 = Agree, 5 = Strongly Agree

† Four of the five raters agreed, but there was one dissenting opinion of "1"

‡ One rater wrote us asking for clarification, noting that subcontracted medical writers have been used in the antidepressant literature, while we described the use of authors directly employed by the pharmaceutical company, a practice known to have occurred in publications on other medication classes such as Cox-2 inhibitors (26). We responded that the rater should decide whether a company employee is similar to a subcontractor when all the details are known. The rater then submitted a rating of "4" (agree) for this question.

Using items from two existing scales, we created a short instrument (convenient to request participation from busy clinicians in a hospital setting) comprising three questions [28] asking respondents to rate how truthful/accurate/credible they found the information presented in the vignettes, and two questions [29] asking respondents to rate how honest and sincere they found the presenter of the information. These questions were rated on a 7-point Likert-type scale. Clinicians were also asked a yes/no question, "If a child you cared about was severely depressed, would you recommend Serovux?". The questions and data are available as a supplemental data file [Additional file 3].

Research participants were degreed personnel working with hospital patients at a metropolitan hospital in the Southwestern United States during Spring 2010, who might be asked by patients for their opinion of a treatment or therapeutic option. A medical social work intern recruited clinicians by approaching them in the hospital face-to-face, either individually or in groups (e.g., staff meetings, Grand Rounds) and asking for their participation in a research study. Participants were not told that the purpose of the research was to examine the impact of COI on research credibility; they were asked only to assist an intern by participating in a research project on "Perceptions of Biomedical Research." Vignettes were distributed so that the COI condition was randomly assigned to participants. Data collection took place between February 1, 2010 and April 1, 2010. The research project was approved by both hospital and University Institutional Review Boards, and each participant provided informed consent.

We had two hypotheses based on the limited published data:

Hypothesis 1: Clinicians randomized to read the COI vignette will rate its information and presenter as less credible than those in the non-COI group.

Hypothesis 2: Clinicians randomized to read the COI vignette will be less likely to recommend the medication than those in the non-COI group.

Power analysis was performed using G*Power version 3.04 [30]. Based on unpublished pilot data, we estimated that we needed 23 participants in each group to detect a 5-point difference (14.29%) on the overall 35-point credibility scale between groups with 95% power. This calculation assumed a standard of deviation of 5 points in each group, using a one-tailed independent t-test. Data analysis was performed with PASW version 18.0 (SPSS Inc., Chicago, IL, USA) and Minitab version 15.0 (Minitab Inc., State College, Pennsylvania, USA).

## Results

59 clinicians were approached and asked to complete the instrument, of which 4 declined to participate and 5 agreed but did not return the instrument. This left 50 completed instruments for analysis, representing an 84.75% response rate.

Of the 50 participants, 17 (34%) were male and 20 (40%) were physicians (see Table 2). There were no missing data for the credibility ratings. Using principal components analysis (PCA), we found that only one component could be extracted and that the 5-question credibility scale was a unidimensional measure of credibility. The Cronbach's alpha for the credibility scale was 0.946. The data were not normally distributed in the No COI group (Shapiro-Wilk test = 0.896, *p *= .018).

**Table 2:** Randomization of subjects by profession and vignette condition

	Physician*	Registered Nurse (Associates Degree)†	Registered Nurse (Advanced degree)‡	Social Worker	Clinical Dietician
**NO COI Condition**	10	3	8	2	1

**COI + GW Condition**	10	8	5	2	1

* Includes 4 medical school students in clinical rotations.

† Groups are imbalanced; the difference is not statistically significant (Pearson χ^2 ^= 2.82, *df = *1*, p = *.093).

‡ Includes Bachelors, Masters, and Doctoral-level nurses.

To test Hypothesis 1, we utilized the nonparametric Mann-Whitney U test to compare overall credibility scores by vignette group (see Table 3). There was a statistically significant difference between groups of 11.00 points (31.42%) on the credibility scale total (95% CI = 6.99 - 15.00; *p < .001*). This is a very large effect size (Cohen's *d *= 1.4), and Hypothesis 1 is supported.

**Table 3:** Credibility ratings by conflict-of-interest condition.*

Outcome Variable	No COI (n = 24)	COI Present (n = 26)	Difference (95% CI)†
Truthful	5.12 ±1.67	3.15 ± 1.41	
	
Accurate	4.83 ±1.46	2.96 ± 1.48	
	
Credible	4.46 ±1.44	2.5 ± 1.63	
	
Honest	5.0 ±1.67	2.81 ± 1.79	
	
Sincere	4.75 ±1.60	3.23 ± 1.75	

Overall Credibility (Scale Total)	24.17 ±6.91	14.65 ± 7.0	11.00 (6.99-15.00)**‡**

* Values are presented as means ± Standard Deviation. COI = Conflict-of-interest, CI = Confidence Interval.

†as calculated through nonparametric Mann-Whitney U test

‡*p *< .001; Cohen's *d *= 1.4, calculated by subtracting the COI present group mean from the No COI group and dividing by the pooled standard deviation.

**Figure 1:**
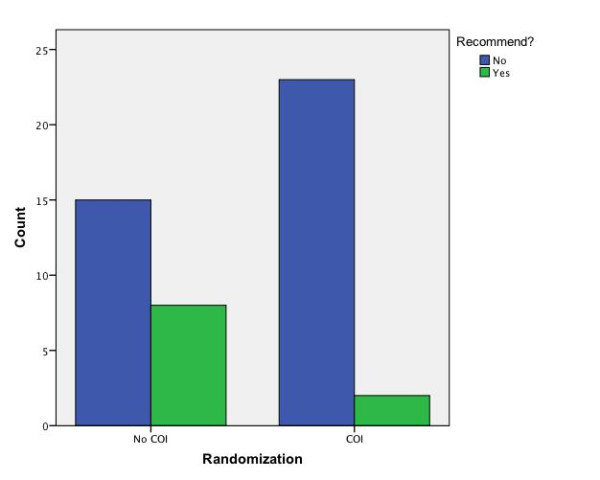
**Clinicians' recommendation of Serovux cross-tabulated by conflict-of-interest condition**.

To test Hypothesis 2, the association between vignette group and recommendation of Serovux was assessed. Two respondents indicated that there was not enough information to make a decision and did not answer this question. Clinicians in the COI group were less likely to recommend Serovux (Pearson χ^2^= 5.21, *df *= 1, *p *= .022). The point estimates for this reduced rate of recommendation were clinically significant (OR = 0.163, 95% CI = .03 - 0.875; RR = 0.709, 95% CI = 0.515 - 0.976) and thus Hypothesis 2 is supported (see Figure 1).

We performed an influence analysis [31] by deleting selected subgroups from the data one at time and then re-running the analyses. Regardless of which group was deleted (men, women, physicians, associates-level nurses, all other nurses), there remained a statistically significant difference in credibility scores between the COI and non-COI groups. Similarly, in all subgroups the Serovux was less likely to be recommended in the COI group. Of note, among the physicians randomized to the COI group, none recommended Serovux.

## Discussion

Hospital-based clinicians (n = 50) who read a fictional vignette describing an antidepressant study found this vignette much less credible when it was accompanied with the disclosure of multiple COI (financial COI, KOL status, and ghostwriting) than when no COI was present. The effect size was very large (a 31.42% difference in credibility scale total as measured by the Mann-Whitney U test; Cohen's *d *= 1.4). Clinicians were also less likely to recommend the medication in the COI condition (OR = 0.163). Influence analyses were performed and the results were resistant to deletion of subgroups.

As Table 3 shows, respondents did not distinguish between the honesty/sincerity of the presenter of the information and the truthfulness/credibility of the study data presented. While there was no content in either vignette suggesting that the data was inaccurate, the presence of multiple COI influenced respondents to perceive the study data as less credible. Past research on consumer trust suggests that trust increases when there is clear accountability and regulation [32]. Since the COI vignette involved a ghostwriting incident with no safeguards and a violation of usual ethical publication norms, this was likely one factor leading respondents to rate the COI vignette as less credible. However, disclosure of financial COI alone lowers perceived credibility [9-11], and given our exploratory design it is not possible to isolate the differential impact of the multiple variables contained in the COI scenario. Some participants may have been aware that selective reporting and suppression of data have been a persistent problem in the antidepressant literature [33,34], sometimes in conjunction with ghostwriting [21], and this may have impacted their credibility ratings.

Interpreting the results of this study largely depends on how realistic the COI vignette is perceived to be. If the COI vignette is seen as a purely theoretical scenario, then the study results may have little application to the real world. However, this does not seem to be the case, as our expert panel found the COI vignette to have excellent face validity, with unanimous agreement that the vignette described a situation similar to real-life. The experts we consulted also reported that the multiple COIs described in our vignette have been common among authors of RCTs in the antidepressant literature, and that psychiatrists reading the antidepressant RCT literature will encounter articles generated in the manner described in our COI vignette. Thus, while our use of multiple COI is a potential drawback, it also represents the 1^st ^published data on how practicing clinicians react to a realistic scenario where the authors simultaneously have multiple COI. Interestingly, one expert characterized the COI vignette as an accurate portrayal of how research on several other classes of medications -- such as mood-stabilizers [35], osteoporosis drugs, and statins -- has been produced. Our findings may thus have application beyond the antidepressant literature.

Our finding that practicing clinicians discount scientific literature when multiple COIs are disclosed may be useful to bioethicists, policymakers, journal editors, and the pharmaceutical industry. For industry, the primary funders of randomized controlled trials, our results suggest that decreased disclosures are preferable. This is interesting in light of new policy developments. New legislation will shortly mandate increased disclosure of industry payments to physicians, and once this information is available on the internet, it will be a simple matter for medical journals to aggregate these data and disclose the exact amounts of financial COI [36], exactly as we did in our vignette. There may also be policy solutions that would increase transparency in authorship [19,37,38]. While these efforts may increase transparency, it will be in the interests of the pharmaceutical industry to oppose their implementation; our results suggest that increased transparency could lead to reduced perceived credibility.

This small study, to our knowledge the first of its kind, should be replicated. The results could be specific to the hospital where the research was conducted, or there could be unobserved confounds. We tested a set of COI (ghostwriting, KOL status and financial COI) against no COI at all, and so we could not separate out the impact of each COI variable individually. A more complex vignette design with more participants could do so, and, through using ANOVA, such an analysis could determine whether there are interaction effects between the various types of COI. The COI information was presented before the clinical content of the vignette, instead of the end, as is the norm in medical journals, and this might have impacted the credibility ratings [11,39]. Our vignette content on KOL status could have been clearer. Despite these limitations, our study provides information on how practicing clinicians perceive research in which multiple COI are disclosed.

## Abbreviations

ANOVA: Analysis of variance; COI: conflict-of-interest; CI: confidence interval; GW: ghostwriting; KOL: key opinion leader; OR: odds ratio; PCA: principal components analysis; RR: relative risk; SNRI: serotonin-norepinephrine reuptake inhibitor; SSRI: selective serotonin reuptake inhibitor.

## Competing interests

JL and JRL are members of Healthy Skepticism, an international non-profit organization dedicated to reducing harm from misleading drug promotion. JRL currently serves as Secretary of Healthy Skepticism.

## Authors' contributions

Conceived the study: JRL. Designed the vignettes: JL JRL. Supervised data collection, analyzed the data, and wrote the first draft: JRL. Participated in writing the paper: JL JRL. Read and approved final version of manuscript: JL JRL.

## Supplementary Material

Additional file 1 **Vignette 1.pdf**. Vignette in which the investigator has no conflicts-of-interest. Click here for file

Additional file 2 **Vignette 2.pdf**. Vignette in which the investigator has multiple conflicts-of-interest. Click here for file

Additional file 3 **Data.xls**. Complete dataset and codebook in Microsoft Excel format. Click here for file
