# Supplementary material for: Knowledge of ghostwriting and financial conflicts-of-interest reduces the perceived credibility of biomedical research
Source: BMC Res Notes. 2011 Jan 31;4:27. doi: 10.1186/1756-0500-4-27 (PMC3037872; doi:10.1186/1756-0500-4-27)
Supplement: Additional file 1 — Vignette 1.pdf. Vignette in which the investigator has no conflicts-of-interest. [file 1756-0500-4-27-S1.PDF]

VIGNETTE 1: NO COI, NO GHOSTWRITING

Dr. Harvey is a researcher and professor of child psychiatry at Northern University, an esteemed academic institution. Dr. Harvey is a very prestigious researcher, and receives funding for his research from the National Institute of Mental Health, a governmental funding agency. He does not receive money from pharmaceutical companies.

Dr. Harvey recently participated in a study of Serovux (an antidepressant) for children. Dr. Harvey treated some of the patients in this study.

With the assistance of a statistician, an editor, and several co-authors, Dr. Harvey co-authored a manuscript reporting the results of the study. Dr. Harvey submitted the manuscript to a prominent medical journal, which reviewed it and agreed to publish it.

Speaking at a press conference about the study, Dr. Harvey said:

"Childhood and adolescent depression is a major health problem, and we need to find effective treatments. So, we did a randomized trial of Serovux- the most rigorous kind of experiment you can do.

We took 200 depressed children, and we randomly prescribed them either Serovux or sugar pills (placebo). The sugar pills looked just like Serovux pills. Neither the children, the parents, or the doctors knew what the child was taking.

We followed the children for eight weeks, and then assessed the children several times. We did clinical interviews to see if they were still depressed, and if so, how severe it was. We used the Children's Depression Rating Scale (C-DRS) which is a scientifically valid way of assessing depression in children.

We found that 62% of the children who were taking Serovux were rated as "much improved" or "very much improved," compared to 34% of children taking placebo. 46% of children taking Serovux had complete remission of their depression, as did 21% of children taking placebo.

We found that side effects were rare- less than 10% of the children experienced any significant side effects.

We conclude that Serovux is a well-tolerated and effective treatment for child and adolescent depression."
